# Time Dysperception Perspective for Acquired Brain Injury

**DOI:** 10.3389/fneur.2013.00217

**Published:** 2014-01-13

**Authors:** Federica Piras, Fabrizio Piras, Valentina Ciullo, Emanuela Danese, Carlo Caltagirone, Gianfranco Spalletta

**Affiliations:** ^1^Neuropsychiatry Laboratory, Department of Clinical and Behavioral Neurology, IRCCS Santa Lucia Foundation, Rome, Italy; ^2^NESMOS Department, University “Sapienza,” Second Faculty of Medicine at Sant’Andrea Hospital, Rome, Italy

**Keywords:** interval timing, cognitive dysfunction, time distortions, brain damage, information processing

## Abstract

Distortions of time perception are presented by a number of neuropsychiatric illnesses. Here we survey timing abilities in clinical populations with focal lesions in key brain structures recently implicated in human studies of timing. We also review timing performance in amnesic and traumatic brain injured patients in order to identify the nature of specific timing disorders in different brain damaged populations. We purposely analyzed the complex relationship between both cognitive and contextual factors involved in time estimation, as to characterize the correlation between timed and other cognitive behaviors in each group. We assume that interval timing is a solid construct to study cognitive dysfunctions following brain injury, as timing performance is a sensitive metric of information processing, while temporal cognition has the potential of influencing a wide range of cognitive processes. Moreover, temporal performance is a sensitive assay of damage to the underlying neural substrate after a brain insult. Further research in neurological and psychiatric patients will clarify whether time distortions are a manifestation of, or a mechanism for, cognitive and behavioral symptoms of neuropsychiatric disorders.

“Time is what prevents everything from happening at once.”

*John Wheeler, physicist*

## Introduction

As both perception and action take place over time and evolve over time, timing is a fundamental component of information processing in the central nervous system. Indeed, our ability to time events in the seconds to minutes range [*interval timing*, Ref. (1, 2)] determines not only the subjective experience of time-in-passing, but also structures our action and cognition allowing us to determine what is happening in our environment and when to respond to events.

Since the representation of time is necessary to appreciate environmental contingencies and estimate predictive relations between events, and between events and responses, timing helps us to interpret reality. Moreover, temporal cognition is a fundamental “*basic unit of ability*” on which other cognitive and behavioral processes are based (3) as for example, complex cognitive functioning is largely dependent on underlying temporal constraints (4). In this sense, temporal processing has the potential of influencing a wide range of cognitive processes [e.g., Ref. (5)].

On the other hand, since interval timing is defined as the ability to perceive, remember, and organize behavior around periods in the range of seconds to minutes, the cognitive apparatus required by this ability include several neuropsychological functions. Supporting cognitive processes comprise allocation of attentional resources to the perception and encoding of incoming temporal information, storage and retrieval of the temporal percept in a long-term memory, and comparison with other percepts in working memory.

Given the inextricable functional interrelation between interval timing and supportive neuropsychological processes, the neuroscientific study of timing can be a model system to study cognitive dysfunction (6). In fact pathophysiological distortions in time might depend on and reflect neuropsychological deficits characteristic of definite neuropsychiatric disorders. Specifically, disrupted timing has been reported in illnesses associated primarily with dopaminergic and fronto-striatal dysfunctions (7, 8) [e.g., Parkinson’s disease (PD), schizophrenia (SZ), Huntington’s disease (HD), attention-deficit hyperactivity disorder (ADHD), see Table 1] (3, 9, 10).

**Table 1:** **Representative studies reporting pathophysiological distortions in time perception and timed performance in neuropsychiatric populations**.

Neuropsychiatric condition	Timing paradigm	Individual differences	Reference
Parkinson’s disease (medicated)	Explicit motor (60) (finger tapping) and non-motor tasks (109) (interval discrimination; duration bisection) in the second and sub-second range	PD groups impaired both in motor timing and time perception in the sub-second and seconds time range	Harrington et al. (60); Smith et al. (109)
Patients with Parkinson’s disease (both on and off medication)	Temporal generalization, bisection, threshold determination, verbal estimation, and a memory for duration task	Stimulus timing performance in patients with PD is relatively normal; small differences may be found on tasks where two stimuli are presented on each trial, possibly as a result of attentional or executive problems	Wearden et al. (110)
	Externally and internally paced finger tapping (111); interval discrimination in the seconds range (112) during functional imaging	Impaired motor timing is consequent to insufficient striatal activity and corticostriatal connectivity (111); the source of impaired time perception in PD is not exclusively temporal, as functional dysfunctions in areas associated with working memory and executive processes were also observed (112)	Jahanshahi et al. (111); Harrington et al. (112)
Patients with pre-symptomatic and symptomatic Huntington’s disease	Explicit motor timing (113) (time estimation) and perceptual time discrimination (113, 114) during fMRI	Even premanifest HD subjects have disrupted time estimation performance which correlates with differences in striatal function; performance declines if timing is embedded in motor processes (113)	Beste et al. (113); Paulsen et al. (114)
Patients affected by schizophrenia	Verbal estimation (115), interval discrimination (116); motor timing [repetitive tapping (116, 117) and time production (118)]	Patients overestimate interval duration when verbally reporting it (115) or during repetitive tapping (117) and underestimate interval duration during time production tasks (118); attentional disturbances (119), working memory deficits (120) or a dysfunction at the decision stage (116) may explain the observed time perception impairment	Tysk (115); Carroll et al. (117); Wahl and Sieg (118); Penney et al. (119); Roy et al. (120); Papageorgiou et al. (116)
Patients with attention deficit hyperactivity disorder	Finger tapping (121–123); interval discrimination (124); interval reproduction (125); interval production (126)	A highly consistent pattern of motor timing abnormalities has been reported both in the sub-second and supra-second intervals scale and in different age groups (121–123). Investigations of duration discrimination, reproduction, and production provide reliable evidence for impairments in ADHD (124–126)	Zelaznick et al. (121); Topla and Tannock (122); Gilden and Marusich (123); Smith et al. (124); Huang et al. (125); Marx et al. (126)

However, since both the dopaminergic system and fronto-striatal circuitry support other cognitive processes such as working memory [e.g., Ref. (11)] and reward based-learning [e.g., Ref. (12), Ref. (13)], either required for accurate timing, questions still remain concerning whether increased timing variability in some neuropsychiatric disorders arises from non-temporal sources or originates from defective interval timing mechanisms. Similarly, multiple lines of evidence (fMRI, lesion, and TMS studies) suggest that prefrontal, frontal, and parietal cortices (14–18), predominantly in the right hemisphere, are crucial for time estimation in the second-to-minute range, although it is not clear whether these brain areas are directly related to time perception, or if they sub serve the non-temporal processes (working memory, recall, and attention) necessary for cognitively controlled time measurements. Indeed, it has been argued (19) that the observed activity in prefrontal and parietal cortices during cognitive controlled temporal tasks could simply reflect the increased sustained attention or working memory demands required by timing longer durations.

Following this line of argument, interval timing can serve a crucial role in models of cognitive dysfunction following brain injury. Actually, since different patterns of brain activity can be elicited by time measurement, and that this is partially dependent upon the nature of the task as well as the participant’s neurological status (20), interval timing could be a valuable construct in neuropathology (6). Specifically, timing performance might be a sensitive metric of cognitive functioning and a reliable assay of damage to the underlying neural substrate (21) [see for example, the hypothesis of a withe matter connectivity damage in mild traumatic brain injury (TBI) deriving from the observation of impaired predictive timing during smooth pursuit eye movements (21)].

Assuming that diverse disorders have their own signature regarding measures of timed behavior, in the present paper we posit that interval timing can be a valid heuristic to explain, in brain damaged populations: (i) the nature of specific cognitive deficits, (ii) the relationships between time estimation and cognition as time judgments are investigated in patients who present memory and attention dysfunctions, and (iii) the essence of such relationship, i.e., whether the correlation between timed and other cognitive behaviors results from specific co-variation of common temporal processes or from coincidental co-variation in the cognitive components (e.g., working memory) shared by the two functions. Indeed, it has been suggested that temporal processing may reflect a “cognitive primitive,” a fundamental neuropsychological process that has a broad influence on cognitive function, constituting part of the functional architecture for cognition (22, 23).

## Isolating Changes in Different Information Processing Stages

The most influential model in the psychological timing literature, the *Scalar Expectancy Theory* (SET) or *internal clock model* (24–26) assumes that at the onset of a to-be-timed event, a switch controlled by attention closes and allows pulses emitted by an internal pacemaker to be collected into an accumulator; the current pulse tally held in working memory is then compared with a previously stored value in reference memory; when the two values match closely enough, a decision rule operates to produce an estimate of time. According to this account, interval timing is highly dependent on other cognitive resources as arousal, vigilance and attention (27–29), working memory, episodic memory, and decision-making (9, 30, 31), such that it may be derivative of these other processes (32, 33). Although the model remains contentious and has a number of competitors, it can easily accommodate individual and pathophysiological differences in timing by adjusting the function of any one of its components. Specifically, considering the plethora of tasks thus far used to experimentally test timing abilities, one can dissect, from the behavioral data gathered in different timing paradigms, the differential contribution of intervening cognitive processes to timing performance. For example, the ability to time short intervals (milliseconds) may be more related to an internal timing mechanism, while longer intervals (seconds) are more related to episodic and working memory (34). Moreover, targeted manipulations can be designed to differentially affect the relative function of the information processing steps described by the SET (35), as the clock stage is influenced by dopaminergic manipulations, and the memory stage by cholinergic manipulations (36, 37).

However, in spite of the fact that contextual factors (i.e., the timing task used) impact temporal performance and the relative contribution of intervening cognitive processes, timing, and time perception in humans (as well as in animals) have striking regularities, as revealed by psychophysical methods used to quantify sensory responses to physical stimuli. Specifically, not only is the intensity of the internal perception linearly related to the magnitude of external stimulation (subjective time increases with physical time), but also increases in the magnitude of a physical stimulus produce proportional increases in the variance of the perception (i.e., it is more difficult to time precisely for longer durations). Deviations from such properties in specific populations are most informative and allow us to make reasonable conjectures as to what mechanisms might be involved in performance differences since for example, damage to and/or pharmacological blockade of corticostriatal circuits impairs the regulation of clock speed as a function of the target duration such that longer intervals will be timed more precisely than shorter intervals, owing to the loss of the scalar source of variability in clock speed (37).

Pertaining to the functional taxonomy of timing, a crucial distinction is made between processes engaged in tasks for which the goal is to provide an overt estimate of elapsed time (explicit timing) as opposed to tasks in which the goal is non-temporal but can, nevertheless, be facilitated by an apparently incidental temporal context [implicit timing, Ref. (38, 39)]. Timing functions are further subcategorized into motor timing (i.e., adjustment of behavior or motor responses to externally or internally defined timeframes, measured in the range of milliseconds and seconds) and perceptual timing (i.e., time estimation and discrimination, also measured in intervals of milliseconds and seconds) (38). Some of these tasks have been originally developed for studying timing in animals [e.g., Ref. (24)] and later extensively applied to humans to test the ways in which different groups (e.g., children, student-age adults, and the elderly) differs [e.g., Ref. (29)].

Actually, most timing paradigms co-measure other non-temporal processes (such as sustained attention or the ability to temporarily hold and manipulate interval representations) depending on the temporal domain they cover. For example, time reproduction relies on working memory functions and the ability to delay a response. Thus, several investigations have tried to examine the relationship between temporal and non-temporal processes. Specifically, time perception varies as a function of selectivity, since attentional resources are limited in time and temporal performance is compromised when attention is overloaded [see Ref. (40) for a meta-analysis on the effect of cognitive load on duration judgments]. It follows that performance in timing tasks is necessarily confounded with attention to time. However, paradigms have been developed to isolate neural networks supporting attention to temporal information from those supporting timing functions *per se* (41) or involved in directing attention to non-temporal stimulus features (42, 43). Temporal attentional orienting can be also modulated pharmacologically, as clonidine, an α2 adrenoceptor agonist which impairs noradrenergic functioning, selectively affects the ability to direct attentional resources to a particular time-point (44), but not time estimation (45). Such observation might be specifically relevant to disorders as ADHD or frontal type dementia in which modulation of noradrenergic release appears to remediate specific attentional functions by attenuating the influence of task-irrelevant information (46).

Also the effective speed of the internal clock can be behaviorally and pharmacologically manipulated, thus affecting judgments of duration: since they directly depend on the number of pulses accumulated, the more pulses that are accumulated, the longer the duration is judged to be, and vice-versa. On the one hand, the rate of subjective time can be changed by producing even slight increases in arousal (e.g., by presenting trains of clicks or a visual flicker) (27). Conversely, as interval timing has been hypothesized to rely on an optimal level of dopaminergic activity in corticostriatal circuits (37), systemic injections of drugs that are believed to promote dopaminergic function (e.g., methamphetamine, cocaine, and nicotine) will determine the underestimation of interval duration (37, 47). This pattern of behavioral change, termed the “clock pattern” (37), is due to the fact that the standard duration representation (e.g., 400 ms) is generated by a normal clock speed while the comparison representations are generated by a putatively faster clock, so that the criterion number of clicks is accumulated in a shorter period (e.g., 350 ms). In a complementary fashion, drugs that are believed to inhibit dopaminergic function (e.g., antipsychotics) produce the overestimation of interval duration due to a decrease in clock speed in the testing phase, so that the criterion amount of clock ticks is accumulated more slowly (37, 48).

Changes in temporal memory and decision processes may also affect event timing and few studies have tried to manipulate the memory stage (49, 50) essentially by increasing temporal memory load (which results in increased variability – i.e., decreased accuracy – in duration judgments). Similarly, a secondary memory task markedly impairs temporal processing of intervals in the range of seconds, since the latter is particularly mediated by memory processes (51). Furthermore, the representation of durations in memory is closely related to decision processes, and a veridical feedback improves the representation of durations in reference memory and modifies the decision criteria. Pharmacological-induced changes in temporal memory are typically produced by cholinergic drugs. Indeed, anticholinesterases such as physostigmine, produce proportional underestimations, and acetylcholine receptor antagonists such as atropine, produce proportional overestimations (37, 47). The hallmark of the specific drug effect on temporal memory is the gradual but long-lasting change in temporal judgments which fails to re-normalize with continued training under the drug (52).

**Figure 1:**
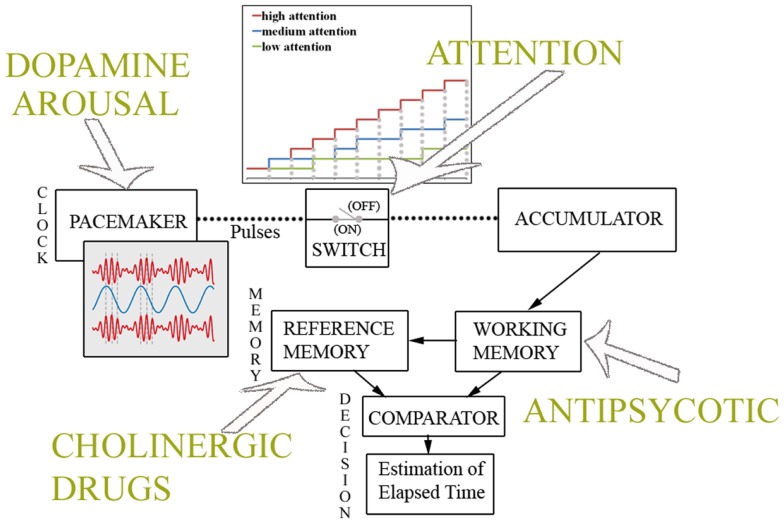
**Cognitive model of time perception**. Information processing model of interval timing as specified by the Scalar Expectancy Theory. As depicted in top panel, the pacemaker speed is affected by dopamine levels and arousal, while attention to time modulates the switch. When attention is high, the estimate of elapsed time grows according to the clock rate, while if attentional resources are overloaded (medium, low attention to time), the switch fluctuates between a closed and an open state, resulting in the underestimation of physical duration. As shown in bottom panel, cholinergic drugs induce changes in temporal memories, whereas antipsychotic medications affect the working memory processes involved in the estimation of elapsed time.

Lastly, since interval timing and working memory are intricately linked in a variety of situations, any behavioral or pharmacological treatment that interferes with maintaining the activation of memory units or attentional processes in working memory may hamper the temporal processing of longer intervals (53). In several pharmacological studies (53, 54), it has been shown that both dopamine antagonists and benzodiazepines that directly affect information processing in working memory, also induce impaired temporal processing of intervals ranging from 1 to 1.4 ms. Figure 1 depicts the cognitive model of time perception as specified by the SET. Pharmacological and cognitive factors affecting different stages of the temporal information processing are also sketched.

## Time Perception in Brain Damaged Populations

### Lesion studies: Identifying critical brain regions

Experimental findings heavily implicate the cerebral cortex, along with the basal ganglia (BG) and cerebellum, in the cognitive processes involved in event timing. In particular, patients with frontal lesions exhibit greater variability [i.e., poorer discrimination, Ref. (55)] when the processing of supra-seconds intervals is required (56, 57). The right prefrontal cortex seems to be involved in longer intervals estimation (14, 16, 58), probably because of its role in other non-temporal processes (sustained attention and working memory) necessary for cognitively controlled time measurements. Timing of longer intervals is also impaired after a lesion, as well as after repetitive transcranial magnetic stimulation (rTMS) functional inhibition (59) to the right parietal lobe (58), thus suggesting that a right prefrontal inferior parietal network is probably engaged in duration estimation (60) especially for longer durations (61). Lesions to the right lateral frontal lobe also prevent the decrease in reaction time that is usually seen in normal subjects when stimulus onset can be anticipated on the basis of the increasing probability of target onset over time (hazard function) (62) or via temporal pre-cues (63). Also patients with right but not left medial temporal lobe resection are impaired in the discrimination of auditory durations in the millisecond range (64), of visual supra-seconds intervals (65) and in the retention of auditory but not visual rhythms (66).

Studies involving subjects with focal lesions to the BG are less conclusive. Indeed, unilateral damage to the BG does not determine timing impairments, at least in motor timing (67), nor abnormalities in temporal orienting tasks in which subjects make deliberate use of temporal pre-cues to anticipate when a target will appear (63). However, even if patients with bilateral lesions to the BG perform relatively well in time estimation tasks, they perform poorly when they are required to maintain rhythmic tapping (68), thus suggesting that BG are essential in reproducing a motor representation of a timed inter-trial-interval (ITI). A bilateral BG dysfunction with significant, disabling clinical signs (68) seems specifically to determine deficits in sub-second timing tasks with a heavy motor component, congruently with accounts that emphasize the role of BG in action.

Also patients with cerebellar damage show marginal (69), non-selective (70) perceptual timing deficits restricted to the sub-second range (55), thus suggesting that the role of cerebellum might be limited to the temporal processing of short durations. Moreover, the cerebellar circuit seems to be vital to performing accurate, rapid rhythmic timing at the fastest intervals (71), as cerebellar patients show increased variability during the synchronization phase at the shortest inter-stimulus-interval (ISI), while patients with neurodegenerative disorders affecting the BG demonstrate less variability but altered accuracy (71, 72). Additionally, cerebellar timing deficits extend to tasks in which patients are required to use the constant spatio-temporal stimulus dynamics to predict its spatio-temporal trajectory (*spatio-temporal prediction*) (73) and show impairments in predictive motor timing, along with difficulties integrating visual stimuli with motor output (74).

**Figure 2:**
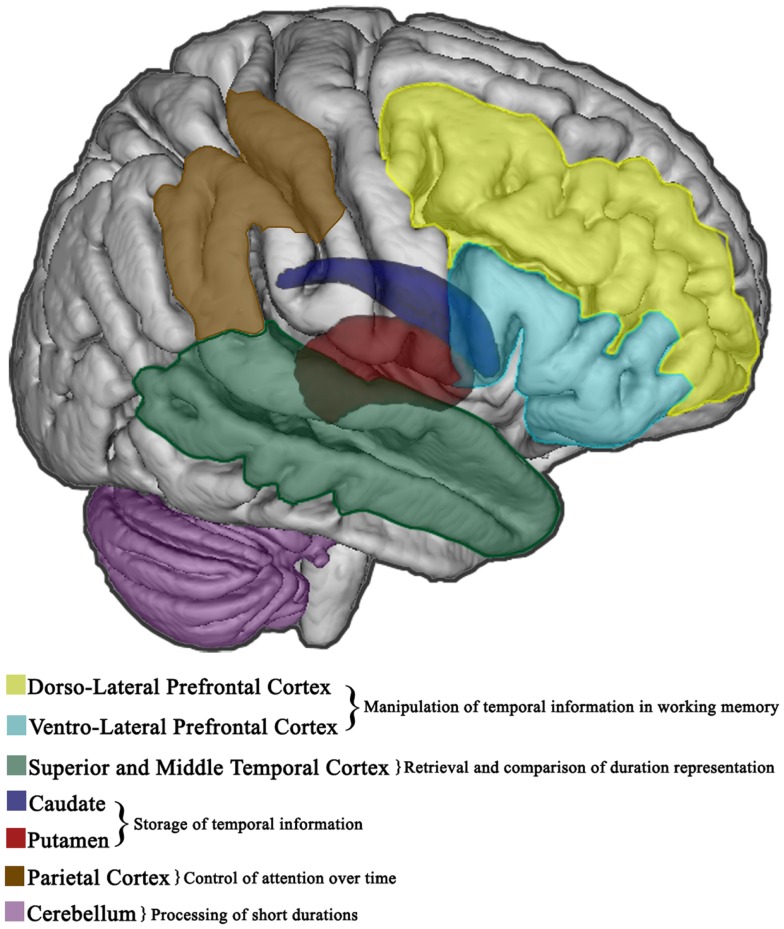
**Explicit timing in the brain**. Cortical and subcortical brain regions involved in the overt estimation of elapsed time (explicit timing). The functional role of different areas in the diverse information processing stages is also specified.

**Figure 3:**
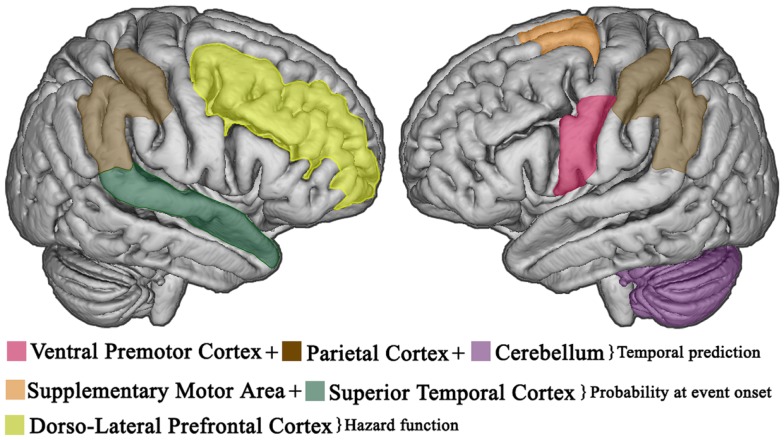
**Temporal expectations in the brain**. Cortical areas involved in implicit, predictive timing. Temporal expectations, based on temporally predictable sequences, are established in left premotor-cerebellar-parietal action circuits. If the event does not occur at the expected delay, the right prefrontal cortex updates the current temporal expectations as a function of elapsed time (hazard function). Once the event occurs, the supplementary motor area and the superior temporal cortex provide an integrated estimate of how expectations evolved over time to improve the accuracy of future prediction.

Taken together such findings indicate that the right prefrontal cortex and BG are essential in explicit duration estimation, while temporal predictions are established in left premotor-cerebellar-parietal circuits (37, 75), and potentially updated on-line in the right prefrontal cortex (76). However, the abovementioned regions have functionally dissociable roles as the right frontal cortex may serve to manipulate temporal information in working memory, while the value is stored in other subcortical regions (i.e., caudate and putamen) (69, 77); subsequent retrieval and comparison of duration representations entail the right temporal cortex (77) which is probably involved in the decision stage (64, 78). Further, the right parietal lobe may have a crucial role in tasks that require the control of attention over time (79). Figures 2 and 3 show the brain regions involved in the explicit and implicit estimation of time intervals as well as their functional role in the processing of temporal information.

### Time perception in amnesic patients: The role of memory processes

Neuropsychological studies in amnesic patients, or those with Korsakoff’s syndrome (80–82) set the opportunity to evaluate the theoretical model of interval timing from the point of view of organic memory disorder. Indeed, since Meck’s first report (78) numerous studies have demonstrated reliable changes in the accuracy and precision of interval timing following a variety of techniques impacting hippocampal function (e.g., transection of the fimbria fornix, lesions of the medial septal area, resection of the temporal lobe, selective lesions of the dorsal hippocampus, and destruction of the entire hippocampus [see Ref. (6) for a review]. Rats and mice with lesions of the hippocampus and related areas, when faced with tasks requiring them to estimate or reproduce a specific duration, respond earlier on average than normal subjects indicating an over estimation/under production of duration due to the complete loss of the ability to hold a representation across a gap in time (6, 78). Similarly, anterograde amnesic patients with severe episodic memory disorders resulting from bilateral temporal lobe lesions, underestimate durations exceeding 15–20 s (82–84), and patients with right temporal lobe lesions are equally affected in the estimations of durations in the second to minutes range (85–87), which presumably involves memory mechanisms. Also reproduction tasks, which require estimating a target duration in order to reproduce it, lead to inaccurate and variable estimations particularly in patients with right temporal lobe lesions (85–87). While short-term and working memory are involved in both the estimation and the reproduction of brief intervals, long-term memory is implicated in timing durations in seconds to minutes range and the under estimation of long durations would reflect a defective retrieval of information in episodic memory, consecutive to medial temporal lesions. Conversely, production tasks which require producing a target duration when given in a conventional unit (seconds or minutes), would entail the comparison with a “typical” duration acquired through life’s experiences and stored in semantic or procedural memory (88). Although this ability might be spared in some amnesic patients (84), it probably requires both right and left medial temporal lobe mechanisms, as patients with either left or right medial temporal lobe lesions overestimated time, suggesting that medial temporal lobe structures are bilaterally involved in the production of intervals in the 1 to 8 min range (89). However, as patients with right medial temporal lobe lesions showed no impairments in a verbal estimation task that also requires the use of chronometric units, patients’ deficits in producing conventional intervals could be partly due to less attention to the passing of time (89).

Moreover, the temporal lobe, and particularly the hippocampus, could participate in the determination of temporal expectancy, which is a continuously updated function of memory, within a fronto-hippocampal circuit modulating the memory stage of the SET. This memory complex, working in tandem with a fronto-striatal circuit which is the basis of the clock stage, would update temporal expectancy on a trial-by-trial basis and provide, once the expected signal or event occurs, an integrated tally of how the expectation evolved over time (90). Owing to dense connections between the hippocampus and the striatum (91), the former could also act as a regulator of the dynamic firing threshold in striatal spiny neurons (7), thus contributing to the encoding of durations. A further possibility is that the hippocampus might be involved, in tandem with the ventral/medial striatal neurons, in the decision-making processes downstream of the clock stage (64, 78), most likely via inhibitory control of action sequences (92).

### Studies in traumatic brain injured patients. Does time perception depend on unimpaired attention and working memory?

Temporal impairment in patients with TBI is expected considering the high incidence of frontal lobe and cognitive dysfunction associated with TBI. Timing dysfunctions have in fact, been documented in patients with focal frontal lesions (55, 56, 70) and attributed to deficits in attention and working memory processes resulting from frontal damage. In view of the fact that slowing of processing speed, reduced attentional capacities, and working memory deficits commonly follow TBI (93), few studies investigated the effect of such disorders on accuracy and precision of duration judgments in TBI (94–99). Some studies employed time reproduction tasks (94, 96, 97) eventually coupled with a time production task (97) and some a time verbal estimation task (94, 98, 99) in which participants are exposed to time intervals and subsequently asked to verbally estimate duration length. The latter is thought to be preferable for studying the sense of time-in-passing (100) since is a measure of how an individuals’ subjective duration of time relates to actual units of time. However, the time durations tested in the abovementioned studies ranged between 4 and 60 s, thus limiting the investigation to supra-second interval timing. Moreover, investigations were mostly conducted on mixed samples of severe and moderate TBI (94, 98, 99), hence making it difficult to discern the impact of brain injury severity on temporal performance. Only two recent studies (95, 101) employed a time discrimination task to examine temporal abilities in TBI patients with short durations. Indeed, compared to time reproduction and production, this task appears to be the best method to detect temporal differences when short intervals are used (102). Additionally, the abovementioned studies were conducted on severe TBI patients in which the prevalence of diffuse axonal injury (103) is likely to result in working memory and attentional deficits, thus affecting temporal performance.

In general, temporal dysfunctions were observed in severe and moderate TBI patients when tested using long intervals, probably exceeding their working memory span (94, 98); likewise, lower accuracy was reported in discriminating a short interval (500 ms as compared to the 1300-ms interval) (101), whereas severe TBI patients and control subjects equally over-produced a brief duration (500 ms) (95). More specifically, variability in time reproduction seems to be correlated with working memory and processing speed performance [at least in patients affected by severe head injury, Ref. (96, 97)], while the variability index for the production task is correlated only with the processing speed measure (97). These findings suggest that severe TBI patients have difficulty in maintaining a stable representation of duration, probably due to impaired sustained attention and working memory. Indeed, despite enduring episodic memory deficits, timing performance in TBI is no longer impaired after a year of recovery (99), implying that other cognitive factors are associated with supra-seconds interval timing. Similarly, TBI patients required working memory and speed of processing abilities to discriminate intervals shorter than a second (101). The observation that no differences were found in a time production task between severe TBI patients and control subjects (101) further suggests that temporal impairment in TBI may not be at the level of the internal clock, as temporal production minimizes the demands on additional cognitive processes, while performance correlates with spontaneous tempo as measured through a tapping task (104).

Lastly, deficits in predictive eye movements during the tracking of a temporally regular stimulus were observed in a group of mild TBI (21) and correlated to measures of working memory and executive control. Through tracking of a temporally predictable target, smooth pursuit eye movements are programed to compensate for visual delays and minimize mismatches between eye and target position and velocity. Such predictive movements are based on an implicit representation of elapsed time (105) and errors in target prediction may be indicative of disorders in the temporal processing component of the task (106). The fact that working memory and executive deficits were correlated with oculomotor measures suggests that parameters of predictive eye movements may be used as a metric of these functions in both the normal population and TBI patients, and as a sensitive assay of damage to the underlying neural connections after TBI (21).

## Relationship between Time Estimation and Cognition

The evidence discussed above emphasizes the complex relationship between both cognitive and contextual factors involved in time estimation. Indeed, this association may be specific either to the time estimation task, or to the range of the target durations employed as for example, episodic memory is differentially involved in reproduction tasks (as opposed to intervals production) and in the estimation of durations exceeding the working memory span. As a case in point, results show that changes in time estimation with aging are related to different cognitive deficits depending on the conditions in which temporal judgments are collected. Shorter reproductions in older adults are better explained by working memory limitations, whereas longer productions in older adults are better explained by slower processing speed (107). Since processing speed is assumed to be related to the clock speed, such observation would suggest that the slowing of information processing with normal aging reflects the slowing of the internal clock rate (108) and would explain age-related changes in duration judgment. Similarly, increased variability in intervals production in TBI patients correlates with processing speed measures, such that temporal performance in this task may not reflect deficits specifically related to timing, but rather to generalized attention and processing speed problems (107). Conversely, increased variability in the reproduction task is related in TBI patients, to updating dysfunctions (101) suggesting that these patients may have difficulties in forming a stable representation of duration due to disorders in working memory. Likewise, lower performance in executive control and working memory correlates with increased variability in measures of implicit timing in mild TBI (21) suggesting that such cognitive factors may influence predictive behaviors based on temporal regularities in stimulus presentation.

Regarding long-term memory, several studies showed that accurate reproduction and discrimination of long durations rely on preserved episodic memory. However, data on duration production tasks are less consistent, as episodic memory should not be necessary in this kind of task, while patients with either left or right medial temporal lobe lesions under-produce intervals in the minutes range (89). Conversely, a semantic/procedural knowledge must be accessed to produce a target duration given in conventional time units and patients with unilateral right temporal lobe resection seem to be selectively impaired in time production abilities (107). Such observation would suggest that the semantic memory involved in the production task depends on the right medial temporal lobe (107); alternatively, patients’ deficits in producing conventional intervals could be partly due to less attention to the passing of time (89). Table 2 summarizes the findings on time distortions in brain damaged patients, outlining the potential mechanism of time dysperception in the considered populations.

**Table 2:** **Summary of pathophysiological distortions in time in neurological and traumatic brain injured patients**.

Timing paradigm	Findings	Intervening cognitive factors
Duration estimation (ms)	Patients with R medial temporal lobe resection more variable in discriminating short intervals (64)	The R medial temporal lobe is involved in controlling the variability of time processing at the level of the decision stage
	Severe TBI patients less accurate when discriminating a short interval (101). The performance significantly correlates with working memory and speed of processing indices	TBI patients seem to refer to cognitively controlled timing to discriminate intervals shorter than a second
Duration estimation (s)	Patients with R prefrontal lesions show robust deficits when discriminating longer durations (56, 57)	The prefrontal cortex sub serves non-temporal processes as sustained attention and working memory required to time long durations
	Patients with bilateral temporal lobe lesions are affected in the estimation of durations in the second to minutes range (85–87)	Amnestic patients underestimate longer durations due to defective retrieval of temporal information in episodic memory
	Underestimation of long intervals after functional inhibition (59) or lesion (58) to the R parietal lobe	A right fronto-parietal network is involved in sustaining attention to time
	Severe and moderate TBI patients significantly different when tested in durations exceeding their working memory span (94, 98)	TBI patients have difficulty in maintaining a stable representation of duration due to impaired sustained attention and working memory
Interval production	Patients with R and L medial temporal lobe lesions over produce intervals in the minutes range (89)	Poorer allocation of attention to the passage of time in patients with medial temporal lesions
	Time pacing at the 1-s tempo is accelerated in some severe TBI patients and slowed down in others (97)	In creased variability in temporal performance due to processing speed problems
	No differences in severe TBI patients when reproducing intervals (101)	Temporal impairment in TBI patients may not be at the level of the internal clock

*The potential mechanism of time dysperception in the different samples is also highlighted*.

## Conclusion

In this paper we surveyed time distortions in clinical populations who have pathophysiological structural alterations in brain areas recently implicated in normal human timing. Given that one can dissect the role of different cognitive processes from the behavioral data gathered in timing paradigms, we could identify the nature of specific timing disorders in different brain damaged populations. We purposely highlighted the interaction between cognitive impairments and loss of accuracy and/or precision in duration judgments, in order to characterize such relationship in each group.

We concluded that while timing variability in TBI patients is not consequent to dysfunctions at the clock stage, but rather related to attentional, working memory and executive functions disorders, medial temporal lobe damage affects the memory component, and possibly the downstream decision-making stage, of the temporal information processing model.

As for the complex relationship between both cognitive and contextual factors involved in time estimation, we posited that the correlation between timed and other cognitive behaviors derive in reproduction and discrimination tasks, from the coincidental co-variation in the cognitive components (e.g., working memory) shared by the two functions. Conversely, variability in production tasks is better explained by variations in processing speed measures (as indexed by reaction time performance), suggesting that time estimation could be a major contributor to reaction time. Indeed, processing speed has been proposed as a “cognitive primitive” participating in several aspects of cognitive functioning, such that variations in the efficiency or effectiveness of specific cognitive processes derive from changes in the speed with which many cognitive operations can be executed. If we assume that the rate of information processing reflects the internal clock rate, then changes in the clock speed due to a neurological disorder would impact the promptitude of executing many different types of processing operations, lastly affecting several cognitive activities.

Following this line of argument, interval timing is a solid construct to study the cognitive effect of a brain insult in specific areas and it has been already posited as a model system to study cognitive aging. Moreover, owing to the extensive overlapping between neural circuits involved in high-level cognitive functions and interval timing, temporal tasks could be used not only as a metric of intellectual functioning, but also as a sensitive assay of damage to the underlying neural substrate.

As time is a fundamental “basic unit of ability” (3) on which other cognitive and behavioral processes are based, future studies should definitively answer the question of whether time distortions are a manifestation of, or a mechanism for, cognitive and behavioral symptoms of brain damage.

## Conflict of Interest Statement

The authors declare that the research was conducted in the absence of any commercial or financial relationships that could be construed as a potential conflict of interest.

## References

[1] Church RM Properties of the internal clock. Ann N Y Acad Sci (1984) 423(1):566–82 10.1111/j.1749-6632.1984.tb23459.x 6588815

[2] Gallistel CR The Organization of Learning. Learning, Development, and Conceptual Change. Cambridge, MA: The MIT Press (1990). 648 p.

[3] Allman MJ Meck WH Pathophysiological distortions in time perception and timed performance. Brain (2012) 135(3):656–77 10.1093/brain/awr210 21921020 PMC3491636

[4] von Steinbüchel N Pöppel E Domains of rehabilitation: a theoretical perspective. Behav Brain Res (1993) 56(1):1–10 10.1016/0166-4328(93)90017-K 8397850

[5] Milner B Corsi P Leonard G Frontal-lobe contribution to recency judgements. Neuropsychologia (1991) 29(6):601–18 10.1016/0028-3932(91)90013-X 1944864

[6] Balci F Meck WH Moore H Brunner D Timing deficits in aging and neuropathology. In: Bizon JL Woods A, editors. Animal Models of Human Cognitive Aging. Aging Medicine. New York, NY: Humana Press (2009). p. 1–41

[7] Matell MS Meck WH Cortico-striatal circuits and interval timing: coincidence detection of oscillatory processes. Brain Res Cogn Brain Res (2004) 21(2):139–70 10.1016/j.cogbrainres.2004.06.012 15464348

[8] Wiener M Lohoff FW Coslett HB Double dissociation of dopamine genes and timing in humans. J Cogn Neurosci (2011) 23(10):2811–21 10.1162/jocn.2011.21626 21261454

[9] Meck WH Neuropsychology of timing and time perception. Brain Cogn (2005) 58(1):1–8 10.1016/j.bandc.2004.09.004 15878722

[10] Teixeira S Machado S Paes F Velasques B Silva J Sanfim A Time perception distortion in neuropsychiatric and neurological disorders. CNS Neurol Disord Drug Targets (2013) 12(5):567–82 10.2174/18715273113129990080 23844680

[11] Blanchard MM Chamberlain SR Roiser J Robbins TW Müller U Effects of two dopamine-modulating genes (DAT1 9/10 and COMT Val/Met) on n-back working memory performance in healthy volunteers. Psychol Med (2011) 41(3):611–8 10.1017/S003329171000098X 21272388

[12] Pierce RC Kumaresan V The mesolimbic dopamine system: the final common pathway for the reinforcing effect of drugs of abuse? Neurosci Biobehav Rev (2006) 30(2):215–38 10.1016/j.neubiorev.2005.04.016 16099045

[13] Adcock RA Thangavel A Whitfield-Gabrieli S Knutson B Gabrieli JDE Reward-motivated learning: mesolimbic activation precedes memory formation. Neuron (2006) 50(3):507–17 10.1016/j.neuron.2006.03.036 16675403

[14] Kagerer FA Wittmann M Szelag E Steinbüchel NV Cortical involvement in temporal reproduction: evidence for differential roles of the hemispheres. Neuropsychologia (2002) 40(3):357–66 10.1016/S0028-3932(01)00111-7 11684169

[15] Lewis PA Miall RC A right hemispheric prefrontal system for cognitive time measurement. Behav Processes (2006) 71(2–3):226–34 10.1016/j.beproc.2005.12.009 16434151

[16] Koch G Oliveri M Carlesimo G Caltagirone C Selective deficit of time perception in a patient with right prefrontal cortex lesion. Neurology (2002) 59(10):1658–9 10.1212/01.WNL.0000032504.45792.8F 12451222

[17] Bueti D Walsh V The parietal cortex and the representation of time, space, number and other magnitudes. Philos Trans R Soc Lond B Biol Sci (2009) 364(1525):1831–40 10.1098/rstb.2009.0028 19487186 PMC2685826

[18] Koch G Oliveri M Caltagirone C Neural networks engaged in milliseconds and seconds time processing: evidence from transcranial magnetic stimulation and patients with cortical or subcortical dysfunction. Philos Trans R Soc Lond B Biol Sci (2009) 364(1525):1907–18 10.1098/rstb.2009.0018 19487193 PMC2685818

[19] Lewis PA Miall RC Brain activation patterns during measurement of sub- and supra-second intervals. Neuropsychologia (2003) 41(12):1583–92 10.1016/S0028-3932(03)00118-0 12887983

[20] Lewis PA Meck WH Time and the sleeping brain. Psychologist (2012) 25(8):594–7

[21] Suh M Kolster R Sarkar R McCandliss B Ghajar J Deficits in predictive smooth pursuit after mild traumatic brain injury. Neurosci Lett (2006) 401(1–2):108–13 10.1016/j.neulet.2006.02.074 16554121

[22] Head D Rodrigue K Kennedy K Raz N Neuroanatomical and cognitive mediators of age-related differences in episodic memory. Neuropsychology (2008) 22(4):491–507 10.1037/0894-4105.22.4.491 18590361 PMC2688704

[23] Foster SM Kisley MA Davis HP Diede NT Campbell AM Davalos DB Cognitive function predicts neural activity associated with pre-attentive temporal processing. Neuropsychologia (2013) 51(2):211–9 10.1016/j.neuropsychologia.2012.09.017 23022080

[24] Gibbon J Church RM, editors. Sources of variance in an information processing theory of timing. Animal Cognition: Proceedings of the Harry Frank Guggenheim Conference, June 2-4, 1982 [held at Columbia University] Hillsdale, NJ: Psychology Press (1984).

[25] Treisman M Temporal discrimination and the indifference interval. Implications for a model of the “internal clock”. Psychol Monogr (1963) 77(13):1–31 10.1037/h0093864 5877542

[26] Creelman CD Human discrimination of auditory duration. J Acoust Soc Am (1962) 34(5):582–93 10.1121/1.1918172

[27] Penton-Voak I Edwards H Percival A Wearden J Speeding up an internal clock in humans? Effects of click trains on subjective duration. J Exp Psychol Anim Behav Process (1996) 22(3):307–20 10.1037/0097-7403.22.3.307 8691161

[28] Wearden J Penton-Voak I Feeling the heat: body temperature and the rate of subjective time, revisited. Q J Exp Psychol (1995) 48(2):129–41 7597195 10.1080/14640749508401443

[29] Wearden JH Pilkington R Carter E ‘Subjective lengthening’ during repeated testing of a simple temporal discrimination. Behav Processes (1999) 46(1):25–38 10.1016/S0376-6357(98)00059-X 24925496

[30] Buhusi CV Meck WH Relative time sharing: new findings and an extension of the resource allocation model of temporal processing. Philos Trans R Soc Lond B Biol Sci (2009) 364(1525):1875–85 10.1098/rstb.2009.0022 19487190 PMC2685821

[31] Buhusi CV Meck WH Relativity theory and time perception: single or multiple clocks? PLoS One (2009) 4(7):e6268 10.1371/journal.pone.0006268 19623247 PMC2707607

[32] Macar F Timing in the new millennium: where are we now? In: Meck WH, editor. Functional and Neural Mechanisms of Interval Timing. Boca Raton, FL: CRC Press (2003). p. 533–40

[33] Zakay D Block R Temporal cognition. Curr Dir Psychol Sci (1997) 6:12–6 10.1111/1467-8721.ep11512604

[34] Ivry RB The representation of temporal information in perception and motor control. Curr Opin Neurobiol (1996) 6(6):851–7 10.1016/S0959-4388(96)80037-7 9000026

[35] Coull JT Hwang HJ Leyton M Dagher A Dopamine precursor depletion impairs timing in healthy volunteers by attenuating activity in putamen and supplementary motor area. J Neurosci (2012) 32(47):16704–15 10.1523/jneurosci.1258-12.2012 23175824 PMC6621775

[36] Rammsayer TH Are there dissociable roles of the mesostriatal and mesolimbocortical dopamine systems on temporal information processing in humans? Neuropsychobiology (1997) 35(1):36–45 10.1159/000119328 9018022

[37] Coull J Cheng R Meck W Neuroanatomical and neurochemical substrates of timing. Neuropsychopharmacology (2011) 36(1):3–25 10.1038/npp.2010.113 20668434 PMC3055517

[38] Coull JT Nobre AC Dissociating explicit timing from temporal expectation with fMRI. Curr Opin Neurobiol (2008) 18(2):137–44 10.1016/j.conb.2008.07.011 18692573

[39] Piras F Coull JT Implicit, predictive timing draws upon the same scalar representation of time as explicit timing. PLoS One (2011) 6(3):e18203 10.1371/journal.pone.0018203 21464972 PMC3064672

[40] Block RA Hancock PA Zakay D How cognitive load affects duration judgments: a meta-analytic review. Acta Psychol (2010) 134(3):330–43 10.1016/j.actpsy.2010.03.006 20403583

[41] Henry MJ Herrmann B Obleser J Selective attention to temporal features on nested time scales. Cereb Cortex (2013):10.1093/cercor/bht240 23978652

[42] Coull JT Nobre AC Where and when to pay attention: the neural systems for directing attention to spatial locations and to time intervals as revealed by both PET and fMRI. J Neurosci (1998) 18(18):7426–35 9736662 10.1523/JNEUROSCI.18-18-07426.1998 PMC6793260

[43] Coull JT Vidal F Nazarian B Macar F Functional anatomy of the attentional modulation of time estimation. Science (2004) 303(5663):1506–8 10.1126/science.1091573 15001776

[44] Coull JT Nobre AC Frith CD The noradrenergic α2 agonist clonidine modulates behavioural and neuroanatomical correlates of human attentional orienting and alerting. Cereb Cortex (2001) 11(1):73–84 10.1093/cercor/11.1.73 11113036

[45] Rammsayer TH Hennig J Haag A Lange N Effects of noradrenergic activity on temporal information processing in humans. Q J Exp Psychol B (2001) 54(3):247–58 10.1080/713932756 11547514

[46] Cinnamon Bidwell L Dew R Kollins S Alpha-2 adrenergic receptors and attention-deficit/hyperactivity disorder. Curr Psychiatry Rep (2010) 12(5):366–73 10.1007/s11920-010-0136-4 20652773 PMC3676929

[47] Meck W Neuropharmacology of timing and time perception. Brain Res Cogn Brain Res (1996) 3(3–4):227–42 10.1016/0926-6410(96)00009-2 8806025

[48] Buhusi CV Meck WH Differential effects of methamphetamine and haloperidol on the control of an internal clock. Behav Neurosci (2002) 116(2):291–7 10.1037/0735-7044.116.2.291 11996314

[49] Wearden JHBS Scalar timing without reference memory? Episodic temporal generalization and bisection in humans. Q J Exp Psychol B (2001) 54(4):289–309 10.1080/02724990042000173 11764836

[50] Jones LAWJH Double standards: memory loading in temporal reference memory. Q J Exp Psychol B (2004) 57(1):55–77 10.1080/02724990344000088 14690849

[51] Rammsayer T Lima S Duration discrimination of filled and empty auditory intervals: cognitive and perceptual factors. Percept Psychophys (1991) 50(6):565–74 10.3758/BF03207541 1780204

[52] Meck WH Choline uptake in the frontal cortex is proportional to the absolute error of a temporal memory translation constant in mature and aged rats. Learn Motiv (2002) 33(1):88–104 10.1006/lmot.2001.1101

[53] Rammsayer TH Neuropharmacological evidence for different timing mechanisms in humans. Q J Exp Psychol B (1999) 52(3):273–86 10.1080/027249999393095 10467900

[54] Lustig C Meck WH Chronic treatment with haloperidol induces deficits in working memory and feedback effects of interval timing. Brain Cogn (2005) 58(1):9–16 10.1016/j.bandc.2004.09.005 15878723

[55] Nichelli P Clark K Hollnagel C Grafman J Duration processing after frontal lobe lesions. Ann N Y Acad Sci (1995) 769(1):183–90 10.1111/j.1749-6632.1995.tb38139.x 8595025

[56] Mangels JA Ivry RB Shimizu N Dissociable contributions of the prefrontal and neocerebellar cortex to time perception. Brain Res Cogn Brain Res (1998) 7(1):15–39 10.1016/S0926-6410(98)00005-6 9714713

[57] Wiener M Coslett HB Disruption of temporal processing in a subject with probable frontotemporal dementia. Neuropsychologia (2008) 46(7):1927–39 10.1016/j.neuropsychologia.2008.01.021 18329055 PMC2494711

[58] Danckert J Ferber S Pun C Broderick C Striemer C Rock S Neglected time: impaired temporal perception of multisecond intervals in unilateral neglect. J Cogn Neurosci (2007) 19(10):1706–20 10.1162/jocn.2007.19.10.1706 17854283

[59] Koch G Oliveri M Torriero S Caltagirone C Underestimation of time perception after repetitive transcranial magnetic stimulation. Neurology (2003) 60(11):1844–6 10.1212/wnl.60.11.1844 12796547

[60] Harrington D Haaland K Hermanowicz N Temporal processing in the basal ganglia. Neuropsychology (1998) 12(1):3–12 10.1037/0894-4105.12.1.3 9460730

[61] N’Diaye K Ragot R Garnero L Pouthas V What is common to brain activity evoked by the perception of visual and auditory filled durations? A study with MEG and EEG co-recordings. Brain Res Cogn Brain Res (2004) 21(2):250–68 10.1016/j.cogbrainres.2004.04.006 15464356

[62] Stuss DT Alexander MP Shallice T Picton TW Binns MA Macdonald R Multiple frontal systems controlling response speed. Neuropsychologia (2005) 43(3):396–417 10.1016/j.neuropsychologia.2004.06.010 15707616

[63] Triviño M Correa Á Arnedo M Lupiáñez J Temporal orienting deficit after prefrontal damage. Brain (2010) 133(4):1173–85 10.1093/brain/awp346 20145048

[64] Melgire M Ragot R Samson S Penney TB Meck WH Pouthas V Auditory/visual duration bisection in patients with left or right medial-temporal lobe resection. Brain Cogn (2005) 58(1):119–24 10.1016/j.bandc.2004.09.013 15878732

[65] Perbal S Ehrlé N Samson S Baulac M Pouthas V Time estimation in patients with right or left medial-temporal lobe resection. Neuroreport (2001) 12(5):939–42 10.1097/00001756-200104170-00015 11303764

[66] Penhune VB Zatorre RJ Feindel WH The role of auditory cortex in retention of rhythmic patterns as studied in patients with temporal lobe removals including Heschls gyrus. Neuropsychologia (1999) 37(3):315–31 10.1016/S0028-3932(98)00075-X 10199645

[67] Aparicio P Diedrichsen J Ivry RB Effects of focal basal ganglia lesions on timing and force control. Brain Cogn (2005) 58(1):62–74 10.1016/j.bandc.2004.09.009 15878727

[68] Coslett HB Wiener M Chatterjee A Dissociable neural systems for timing: evidence from subjects with basal ganglia lesions. PLoS One (2010) 5(4):e10324 10.1371/journal.pone.0010324 20428244 PMC2859062

[69] Harrington DL Lee RR Boyd LA Rapcsak SZ Knight RT Does the representation of time depend on the cerebellum? Effect of cerebellar stroke. Brain (2004) 127(3):561–74 10.1093/brain/awh065 14711883

[70] Casini L Ivry R Effects of divided attention on temporal processing in patients with lesions of the cerebellum or frontal lobe. Neuropsychology (1999) 13(1):10–21 10.1037/0894-4105.13.1.10 10067771

[71] Claassen DO Jones CRG Yu M Dirnberger G Malone T Parkinson M Deciphering the impact of cerebellar and basal ganglia dysfunction in accuracy and variability of motor timing. Neuropsychologia (2013) 51(2):267–74 10.1016/j.neuropsychologia.2012.09.018 23084982

[72] Jones CRG Claassen D Yu M Spies JR Malone T Dirnberger G Modelling accuracy and variability of motor timing in treated and untreated Parkinson’s disease and healthy controls. Front Integr Neurosci (2011) 5:81 10.3389/fnint.2011.00081 22207839 PMC3245650

[73] Beudel M Renken R Leenders KL de Jong BM Cerebral representations of space and time. Neuroimage (2009) 44(3):1032–40 10.1016/j.neuroimage.2008.09.028 18951984

[74] Bareš M Lungu O Husárová I Gescheidt T Predictive motor timing performance dissociates between early diseases of the cerebellum and Parkinson’s disease. Cerebellum (2010) 9(1):124–35 10.1007/s12311-009-0133-5 19851820

[75] O’Reilly JX Mesulam MM Nobre AC The cerebellum predicts the timing of perceptual events. J Neurosci (2008) 28(9):2252–60 10.1523/jneurosci.2742-07.2008 18305258 PMC6671847

[76] Vallesi A Mussoni A Mondani M Budai R Skrap M Shallice T The neural basis of temporal preparation: Insights from brain tumor patients. Neuropsychologia (2007) 45(12):2755–63 10.1016/j.neuropsychologia.2007.04.017 17544014

[77] Coull JT Nazarian B Vidal F Timing, storage, and comparison of stimulus duration engage discrete anatomical components of a perceptual timing network. J Cogn Neurosci (2008) 20(12):2185–97 10.1162/jocn.2008.20153 18457512

[78] Meck WH Attentional bias between modalities: effect on the internal clock, memory, and decision stages used in animal time discrimination. Ann N Y Acad Sci (1984) 423(1):528–41 10.1111/j.1749-6632.1984.tb23457.x 6588813

[79] Battelli L Pascual-Leone A Cavanagh P The ‘when’ pathway of the right parietal lobe. Trends Cogn Sci (2007) 11(5):204–10 10.1016/j.tics.2007.03.001 17379569 PMC3613278

[80] Mimura M Kinsbourne M O’Connor M Time estimation by patients with frontal lesions and by Korsakoff amnesics. J Int Neuropsychol Soc (2000) 6(5):517–28 10.1017/S1355617700655017 10932471

[81] Shaw C Aggleton JP The ability of amnesic subjects to estimate time intervals. Neuropsychologia (1994) 32(7):857–73 10.1016/0028-3932(94)90023-X 7936168

[82] Williams JM Medwedeff CH Haban G Memory disorder and subjective time estimation. J Clin Exp Neuropsychol (1989) 11(5):713–23 10.1080/01688638908400927 2808660

[83] Kinsbourne M Hicks RE The extended present: evidence from time estimation by amnesics and normals. In: Shallice GVT, editor. Neuropsychological Impairments of Short-Term Memory. New York, NY: Cambridge University Press (1990). p. 319–30

[84] Perbal S Pouthas V Van der Linden M Time estimation and amnesia: a case study. Neurocase (2000) 6(4):347–56 10.1080/13554790008402782

[85] Basso G Nichelli P Frassinetti F di Pellegrino G Time perception in a neglected space. Neuroreport (1996) 7(13):2111–4 10.1097/00001756-199609020-00009 8930969

[86] Rubia K Schuri U Cramon DYV Poeppel E Time estimation as a neuronal network property: a lesion study. Neuroreport (1997) 8(5):1273–6 10.1097/00001756-199703240-00043 9175128

[87] Vidalaki VN Ho MY Bradshaw CM Szabadi E Interval timing performance in temporal lobe epilepsy: differences between patients with left and right hemisphere foci. Neuropsychologia (1999) 37(9):1061–70 10.1016/S0028-3932(98)00155-9 10468369

[88] Perbal-Hatif S A neuropsychological approach to time estimation. Dialogues Clin Neurosci (2012) 14(4):425–32 23393418 10.31887/DCNS.2012.14.4/sphatif PMC3553566

[89] Noulhiane M Pouthas V Hasboun D Baulac M Samson S Role of the medial temporal lobe in time estimation in the range of minutes. Neuroreport (2007) 18(10):1035–8 10.1097/WNR.0b013e3281668be1 17558291

[90] Coull JT Neural substrates of mounting temporal expectation. PLoS Biol (2009) 7(8):e1000166 10.1371/journal.pbio.1000166 19652699 PMC2711332

[91] Lee AS Duman RS Pittenger C A double dissociation revealing bidirectional competition between striatum and hippocampus during learning. Proc Natl Acad Sci U S A (2008) 105(44):17163–8 10.1073/pnas.0807749105 18955704 PMC2579395

[92] Yin B Troger A Exploring the 4th dimension: hippocampus, time, and memory revisited. Front Integr Neurosci (2011) 5:36 10.3389/fnint.2011.00036 21886612 PMC3154297

[93] Hartikainen KM Wäljas M Isoviita T Dastidar P Liimatainen S Solbakk A-K Persistent symptoms in mild to moderate traumatic brain injury associated with executive dysfunction. J Clin Exp Neuropsychol (2010) 32(7):767–74 10.1080/13803390903521000 20198531

[94] Meyers CA Levin HS Temporal perception following closed head injury: relationship of orientation and attention span. Neuropsychiatry Neuropsychol Behav Neurol (1992) 5(1):28–32

[95] Mioni G Mattalia G Stablum F Time perception in severe traumatic brain injury patients: a study comparing different methodologies. Brain Cogn (2013) 81(3):305–12 10.1016/j.bandc.2012.12.005 23395855

[96] Mioni G Stablum F McClintock SM Cantagallo A Time-based prospective memory in severe traumatic brain injury patients: the involvement of executive functions and time perception. J Int Neuropsychol Soc (2012) 18(4):697–705 10.1017/S1355617712000306 22433779

[97] Perbal S Couillet J Azouvi P Pouthas V Relationships between time estimation, memory, attention, and processing speed in patients with severe traumatic brain injury. Neuropsychologia (2003) 41(12):1599–610 10.1016/S0028-3932(03)00110-6 12887985

[98] Schmitter-Edgecombe M Rueda AD Time estimation and episodic memory following traumatic brain injury. J Clin Exp Neuropsychol (2008) 30(2):212–23 10.1080/13803390701363803 18938673

[99] Anderson JW Schmitter-Edgecombe M Recovery of time estimation following moderate to severe traumatic brain injury. Neuropsychology (2011) 25(1):36–44 10.1037/a0020333 20919767 PMC3018715

[100] Kinsbourne M The role of memory in estimating time: a neuropsychological analysis. In: Connor L Obler L, editors. Neurobehavior of Language and Cognition. Norwell, MA: Springer (2002). p. 315–23

[101] Mioni G Stablum F Cantagallo A Time discrimination in traumatic brain injury patients. J Clin Exp Neuropsychol (2012) 35(1):90–102 10.1080/13803395.2012.755151 23259647

[102] Rammsayer TH Ageing and temporal processing of durations within the psychological present. Eur J Cogn Psychol (2001) 13(4):549–65 10.1080/09541440125713

[103] Skandsen T Kvistad KA Solheim O Strand IH Folvik M Vik A Prevalence and impact of diffuse axonal injury in patients with moderate and severe head injury: a cohort study of early magnetic resonance imaging findings and 1-year outcome. J Neurosurg (2010) 113(3):556–63 10.3171/2009.9.JNS09626 19852541

[104] Baudouin A Vanneste S Isingrini M Pouthas V Differential involvement of internal clock and working memory in the production and reproduction of duration: a study on older adults. Acta Psychol (2006) 121(3):285–96 10.1016/j.actpsy.2005.07.004 16139783

[105] Fukushima K Fukushima J Warabi T Barnes GR Cognitive processes involved in smooth pursuit eye movements: behavioral evidence, neural substrate and clinical correlation. Front Syst Neurosci (2013) 7:4 10.3389/fnsys.2013.00004 23515488 PMC3601599

[106] Badler J Lefèvre P Missal M Anticipatory pursuit is influenced by a concurrent duration reproduction task. J Vis (2008) 8(6):672 10.1167/8.6.672 19156986

[107] Pouthas V Perbal S Time perception depends on accurate clock mechanisms as well as unimpaired attention and memory processes. Acta Neurobiol Exp (Wars) (2004) 64(3):367–85 15283479 10.55782/ane-2004-1520

[108] Wearden J Wearden A Rabbitt P Age and IQ effects on stimulus and response timing. J Exp Psychol Hum Percept Perform (1997) 23:962–79 10.1037/0096-1523.23.4.962

[109] Smith JG Harper DN Gittings D Abernethy D The effect of Parkinson’s disease on time estimation as a function of stimulus duration range and modality. Brain Cogn (2007) 64(2):130–43 10.1016/j.bandc.2007.01.005 17343966

[110] Wearden JH Smith-Spark JH Cousins R Edelstyn NMJ Cody FWJ O’Boyle DJ Stimulus timing by people with Parkinson’s disease. Brain Cogn (2008) 67(3):264–79 10.1016/j.bandc.2008.01.010 18329150

[111] Jahanshahi M Jones CRG Zijlmans J Katzenschlager R Lee L Quinn N Dopaminergic modulation of striato-frontal connectivity during motor timing in Parkinson’s disease. Brain (2010) 133(3):727–45 10.1093/brain/awq012 20305278

[112] Harrington DL Castillo GN Greenberg PA Song DD Lessig S Lee RR Neurobehavioral mechanisms of temporal processing deficits in Parkinson’s disease. PLoS One (2011) 6(2):e17461 10.1371/journal.pone.0017461 21364772 PMC3045463

[113] Beste C Saft C Andrich J Müller T Gold R Falkenstein M Time processing in Huntington’s disease: a group-control study. PLoS One (2007) 2(12):e1263 10.1371/journal.pone.0001263 18060059 PMC2094403

[114] Paulsen JS Zimbelman JL Hinton SC Langbehn DR Leveroni CL Benjamin ML fMRI biomarker of early neuronal dysfunction in presymptomatic Huntington’s disease. AJNR Am J Neuroradiol (2004) 25(10):1715–21 15569736 PMC8148746

[115] Tysk LPMSJ Time estimation by healthy subjects and schizophrenic patients: a methodological study. Percept Mot Skills (1983) 56(3):983–8 10.2466/pms.1983.56.3.983 6877984

[116] Papageorgiou C Karanasiou IS Kapsali F Stachtea X Kyprianou M Tsianaka EI Temporal processing dysfunction in schizophrenia as measured by time interval discrimination and tempo reproduction tasks. Prog Neuropsychopharmacol Biol Psychiatry (2013) 40(0):173–9 10.1016/j.pnpbp.2012.07.017 23367507

[117] Carroll CA O’Donnell BF Shekhar A Hetrick WP Timing dysfunctions in schizophrenia span from millisecond to several-second durations. Brain Cogn (2009) 70(2):181–90 10.1016/j.bandc.2009.02.001 19282082

[118] Wahl O Sieg D Time estimation among schizophrenics. Percept Mot Skills (1980) 50(2):535–41 10.2466/pms.1980.50.2.535 7375306

[119] Penney TB Meck WH Roberts SA Gibbon J Erlenmeyer-Kimling L Interval-timing deficits in individuals at high risk for schizophrenia. Brain Cogn (2005) 58(1):109–18 10.1016/j.bandc.2004.09.012 15878731

[120] Roy M Grondin S Roy M-A Time perception disorders are related to working memory impairment in schizophrenia. Psychiatry Res (2012) 200(2–3):159–66 10.1016/j.psychres.2012.06.008 22862910

[121] Zelaznik HN Vaughn AJ Green JT Smith AL Hoza B Linnea K Motor timing deficits in children with attention-deficit/hyperactivity disorder. Hum Mov Sci (2012) 31(1):255–65 10.1016/j.humov.2011.05.003 21852012 PMC3223335

[122] Toplak ME Tannock R Tapping and anticipation performance in attention deficit hyperactivity disorder. Percept Mot Skills (2005) 100(3):659–75 10.2466/pms.100.3.659-675 16060425

[123] Gilden DL Marusich LR Contraction of time in attention-deficit hyperactivity disorder. Neuropsychology (2009) 23(2):265–9 10.1037/a0014553 19254099

[124] Smith A Taylor E Warner Rogers J Newman S Rubia K Evidence for a pure time perception deficit in children with ADHD. J Child Psychol Psychiatry (2002) 43(4):529–42 10.1111/1469-7610.00043 12030598

[125] Huang J Yang B-R Zou X-B Jing J Pen G McAlonan GM Temporal processing impairment in children with attention-deficit-hyperactivity disorder. Res Dev Disabil (2012) 33(2):538–48 10.1016/j.ridd.2011.10.021 22119703

[126] Marx I Hübner T Herpertz S Berger C Reuter E Kircher T Cross-sectional evaluation of cognitive functioning in children, adolescents and young adults with ADHD. J Neural Transm (2010) 117(3):403–19 10.1007/s00702-009-0345-3 19953279

